# Challenges and Opportunities of Big Data in Health Care: A Systematic Review

**DOI:** 10.2196/medinform.5359

**Published:** 2016-11-21

**Authors:** Clemens Scott Kruse, Rishi Goswamy, Yesha Raval, Sarah Marawi

**Affiliations:** ^1^ School of Health Administration Texas State University San Marcos, TX United States

**Keywords:** big data, analytics, health care, human genome, electronic medical record

## Abstract

**Background:**

Big data analytics offers promise in many business sectors, and health care is looking at big data to provide answers to many age-related issues, particularly dementia and chronic disease management.

**Objective:**

The purpose of this review was to summarize the challenges faced by big data analytics and the opportunities that big data opens in health care.

**Methods:**

A total of 3 searches were performed for publications between January 1, 2010 and January 1, 2016 (PubMed/MEDLINE, CINAHL, and Google Scholar), and an assessment was made on content germane to big data in health care. From the results of the searches in research databases and Google Scholar (N=28), the authors summarized content and identified 9 and 14 themes under the categories *Challenges* and *Opportunities*, respectively. We rank-ordered and analyzed the themes based on the frequency of occurrence.

**Results:**

The top challenges were issues of data structure, security, data standardization, storage and transfers, and managerial skills such as data governance. The top opportunities revealed were quality improvement, population management and health, early detection of disease, data quality, structure, and accessibility, improved decision making, and cost reduction.

**Conclusions:**

Big data analytics has the potential for positive impact and global implications; however, it must overcome some legitimate obstacles.

## Introduction

### Rationale

Big data analytics offers promise in many business sectors, and health care is looking at big data to provide answers to many age-related issues, particularly dementia and chronic disease management. This systematic review explores the depth of big data analytics since 2010 and identifies both challenges and opportunities associated with big data in health care. The review follows the standard set by Preferred Reporting Items for Systematic Reviews and Meta-analysis (2009) [1].

Big data is commonly defined through the 4 Vs: volume (scale or quantity of data), velocity (speed and analysis of real-time or near-real-time data), variety (different forms of data, often from disparate data sources), and veracity (quality assurance of the data). The first 3 Vs are found in most literature [2,3], and the fourth V is a goal [4].

As of 2012, about 2.5 exabytes of data are created each day; Walmart can collect up to 2.5 petabytes of customer-related data per hour [2]. The industry of health care produces and collects data at a staggering speed, but different electronic health records (EHRs) collect data in different structures: structured, unstructured, and semistructured. This variety can pose difficulty when seeking veracity or quality assurance of the data. The EHRs can provide a rich source of data, ripe for analysis to increase our understanding of disease mechanisms, as well as better and personalized health care, but the data structures pose a problem to standard means of analysis [5].

There are several large sources for big data in health care: genomics, EHR, medical monitoring devices, wearable video devices, and health-related mobile phone apps. Approximately 483 studies on genomics are registered with the US Department of Health and Human Services; these studies are being conducted in 9 countries, and they all use portions of the data from the Human Genome Project [6]. The EHR, being adopted in many countries, offers a source of data the depth of which is almost inconceivable. About 500 petabytes of data was generated by the EHR in 2012, and by 2020, the data will reach 25,000 petabytes [7]. The EHR can collect data from other monitoring devices, but the continuous data streams are not consistently saved in the longitudinal record.

The decrease in the cost of storage has enabled an exponential distribution of data collection, but the ability to analyze this quantity of data is the center of gravity for “big data” in health care. In the United States, financial incentives offered for the “meaningful use” of health information technology has spurred growth in the adoption of the EHR and other enabling health-related technology since 2009.

Health information systems show great potential in improving the efficiency in the delivery of care, a reduction in overall costs to the health care system, as well as a marked increase in patient outcomes [8]. The US government has allocated billions of dollars to help the country’s health care market realize some of these efficiencies and savings. Specific provisions of the Health Information Technology for Economic and Clinical Health (HITECH), part of the American Recovery and Reinvestment Act, acknowledge the importance of IT in the delivery of health care within the United States [9]. The Act allocates approximately US $17.2 billion in incentives for the adoption and meaningful use of health information technology, part of which involves the participation in the electronic exchange of clinical information. In 2010, the Congress passed the Health Information Exchange (HIE) Challenge Grant Program, which contributed about US $547.7 million to state HIE programs [10].

With the implementation of this legislation as well as the technologies associated with it, it is imperative to effectively organize and process the ever-increasing quantity of data that is digitally collected and stored within health care organizations. Other industries such as astronomy, retail, search engines, and politics have developed advanced data-handling capabilities to convert data into knowledge. Health care needs to follow their lead so that decisions regarding organizational objectives and goals can be met [4,11,12]. This evolutionary process of data management is collectively known as big data, and it is essential to the future of adoption and management of health information technology [13].

### Objectives

The purpose of this systematic review is to objectively review articles and studies published in academic journals in order to compile a list of challenges and opportunities faced by big data analytics in health care in the United States. Particular emphasis was paid to age-related applications of big data.

## Methods

### Eligibility Criteria

Articles and studies were eligible for analysis if they were published between 2010 and 2015, published in academic journals, and published in English. The researchers chose a range from 2010 to 2015 for two reasons: HITECH was passed in 2009, and it appeared that a blossom of research and other articles seemed to occur in 2010. We focused on academic journals for their peer-review quality and to decrease the chance of selecting something about big data published from a noncredible source.

### Information Sources

**Figure 1:**
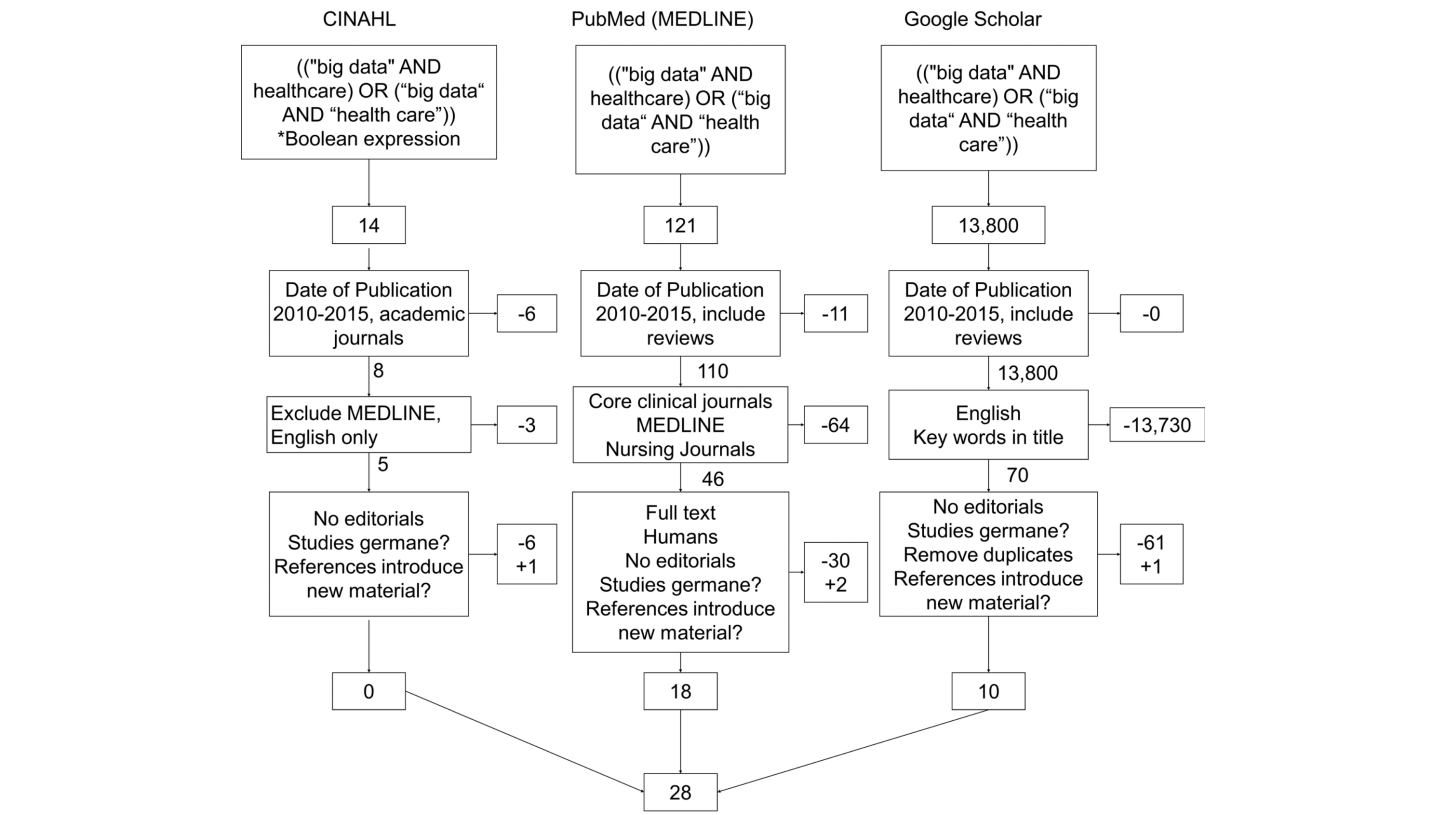
Literature review process with inclusion and exclusion criteria.

A combination of key terms from Medical Subject Headings (MeSH) and Boolean operators were combined and used in 2 common research databases, CINAHL and PubMed, and combined with a general search from Google Scholar (see Figure 1) in January 2016.

These terms were chosen not only because they are the focus of the review, but also because they were identified in the initial research into the definition of big data.

### Search

The following search string was used in all 3 searches: ((“big data” AND healthcare) OR (“big data” AND “health care”)). This search string was used in CINAHL, PubMed (MEDLINE), and Google Scholar. In the 2 research databases, our team was able to restrict the search to academic journals (including other systematic reviews). MEDLINE was excluded in CINAHL because it was already captured in PubMed. Google Scholar creates difficulty for searches because of its severe limit of filters typically associated with academic research. The initial 13,935 results were limited by restricting dates to the last 5 years, limiting results to academic journals and MEDLINE, and in Google Scholar by restricting the keyword search to titles. The result from the filters ended with 121 articles to review.

### Study Selection

Through group research and a series of consensus meetings, researchers were trained to identify articles germane to this review and to recommend elimination of all others. A shared spreadsheet was used by the research team to parse through the list of articles. Researchers read all articles in their entirety. A total of 97 articles were eliminated due to various exclusion criteria (not germane to big data or health care, editorial only, not an academic journal, or duplicate from another search), and 4 additional articles were identified from the references of the 24 that remained. The group of reviewers made these rejections or additional recommendations through a series of consensus meetings where we met to discuss their recommendations and consensus was reached through discussion. A total of 28 articles remained in the final review.

### Data Collection Process and Identification of Summary Measures

Each article was reviewed by at least two authors to identify the relevant points. All reviewers used a spreadsheet template to summarize their key observations from each article. One team member combined the spreadsheets into one and shared it once again. Reviewers held one more consensus meeting to discuss their findings. From this meeting, trends were identified, and from those trends, inferences were made.

### Additional Analysis

From the list of observations, reviewers were able to identify some common threads that emerged as challenges and opportunities in health care that permeated multiple articles. Separate tables were created to group the threads, and from each of these tables, common themes were identified. These common themes only emerged when reviewers combined their observations. These themes were tabulated and counted for additional analysis.

## Results

### Study Selection

As depicted in Figure 1, 935 articles resulted from the initial search. Filters such as data published (2010-2015), academic journals, and English language were implemented to reduce the range to what was being studied. Reviewers agreed to eliminate editorials and focus on those articles that studied big data, as described in the Introduction section of this manuscript. At the end of the search process, only 28 remained. The articles reviewed for this study ranged from 2012 to 2015. The majority of the literature chosen for this paper was published in 2014 (15/28, 54%), and a minority was published in 2015 (2/28, 7%); the latter was most likely due to the early part of the year when the search was conducted.

### Synthesis of Results

Multiple reviewers read each article in its entirety. Articles were included or excluded based on the criteria illustrated in Figure 1. All articles included in the analysis were sorted by date and are listed in Multimedia Appendix 1.

A study catalog number was assigned to each article to simplify the analysis. Researchers summarized the main points of each article for further analysis.

### Additional Analysis

Through the combination of observations, reviewers identified common threads (challenges and opportunities) and themes from each thread. Themes were organized into affinity diagrams (Tables 1 and 2), compared, and discussed among researchers.

#### Challenges for Big Data in Health Care

Nine themes emerged under the category of challenges: data structure, security, data standardization, data storage and transfers, managerial issues such as governance and ownership, lack of skill of data analysts, inaccuracies in data, regulatory compliance, and real-time analytics. Examples for each theme are provided in Table 1. A total of 60 observations were made for challenges.

**Table 1:** Themes associated with challenges for big data in health care.

Themes	Examples	Number of articles (n)	Articles themes appeared in	% of total articles (N=28)
Data structure	Fragmented data	17	1, 2, 7-9, 12, 14-19, 22, 25-28	61%
	Incompatible formats			
	Heterogeneous data			
	Raw and unstructured datasets			
	Large volumes			
	High variety and velocity			
	Lack of transparency			
Security	Privacy	14	2, 4, 7-9, 12, 13, 17, 21, 22, 25-28	50%
	Confidentiality			
	Data duplication			
	Integrity			
Data standardization	Limited Interoperability	11	4, 5, 7-9, 11, 12, 15, 16, 22, 25	39%
	Data acquisition and cleansing			
	Global sharing			
	Terminology			
	Language barriers			
Storage and transfers	Expensive to store	8	1, 4, 7, 12, 22, 26, 28	28%
	Transfer from one place to other			
	Store electronic data			
	Securely extract, transmit, and process			
Managerial issues	Governance issues	4	2, 8, 14, 22	14%
	Ownership issues			
Lack of skill	Untrained workers	3	5, 9, 14	11%
Inaccuracies	Inconsistences	1	9	4%
	Lack of precision			
	Data timeliness			
Regulatory compliance	Legal concerns	1	13	4%
Real-time analytics	Real-time analytics	1	9	4%

The 4 Vs appear in multiple places under the Challenges category. Volume and variety are seen by name under the theme of Data structure. Variety is also implied in the same theme, but listed as Incompatible formats, as well as Raw and unstructured datasets. Variety can also be inferred from the theme of Data standardization, listed as Limited interoperability. Velocity is seen in the theme Real-time analytics. Veracity is seen under the theme of Data Standardization, but listed as Data acquisition and cleansing, Terminology, and Language barriers. It is also inferred in the theme Inaccuracies listed as Inconsistencies and Lack of precision.

##### Data Structure Issues

Issues related to data structure were addressed in the majority of the papers reviewed for this study. It is essential that the key functions of data processing are supported by the applications of big data [13]. Big data applications should be user-friendly, transparent, and menu-driven [13,14]. The majority of data in health care is unstructured, such as from natural language processing [12]. It is often fragmented, dispersed, and rarely standardized [12,13,15-21]. It is no secret that the EHRs do not share well across organizational lines, but with unstructured data, even within the same organization, unstructured data is difficult to aggregate and analyze. It is no wonder that 61% of the articles analyzed listed this as a concern; big data analytics will need to address this large challenge.

Research data within the health care sector is more heterogeneous than the research data produced within other research fields [3,5,12]. Data from both research and public health is often produced in large volumes [15,22,23]. Another structure-related issue results from the changing health care fee-for-service care model [4]. Finally, big data will need to address issues with the transparency of metadata [16,24].

##### Security Issues

There are considerable privacy concerns regarding the use of big data analytics, specifically in health care given the enactment of Health Insurance Portability and Accountability Act (HIPAA) legislation [15]. Data that is made available on open source is freely available and, hence, highly vulnerable [12,13,18,20]. Further, due to the sensitivity of health care data, there are significant concerns related to confidentiality [25,26]. Moreover, this information is centralized, and as such, it is highly vulnerable to attacks [25]. For these reasons, enabling privacy and security is very important, as illustrated by a frequency of mention in 50% of the literature reviewed.

##### Data Standardization Issues

Although the EHRs share data within the same organization, intra-organizational, EHR platforms are fragmented, at best. Data is stored in formats that are not compatible with all applications and technologies [13,22]. This lack of data standardization also causes problems in transfer of that data [5,25]. It complicates data acquisition and cleansing [5,25,26]. About 39% of the literature mentioned this challenge.

Limited interoperability poses a large challenge for big data, as data is rarely standardized [12,13,16,22]. This leaves big data to face issues related to the acquisition and cleansing of data into a standardized format to enable analysis and global sharing [13,17,23,25,27]. With globalization of data, big data will have to deal with a variety of standards, barriers of language, and different terminologies.

##### Storage and Transfers

Data generation is inexpensive compared with the storage and transfer of the same. Once data is generated, the costs associated with securing and storing them remain high [25]. Costs are also incurred with transferring data from one place to another as well as analyzing it [14,21,22]. Some researchers have been able to combine the themes of Data structure and Storage and transfers when they illustrate how structured data can be easily stored, queried, analyzed, and so forth, but unstructured data is not as easily manipulated [13]. Cloud-based health information technology has the additional layer of security associated with the extraction, transformation, and loading of patient-related data [27]. The use of big data should address issues related to increased expenditures as well as the transmittance of secure or insecure information. About 28% of the literature mentioned this challenge.

##### Managerial Issues

Data governance will need to move up on the priority list of organizations, and it should be treated as a primary asset instead of a by-product of the business [15]. Data ownership and data stewardship should create new roles in business that consider big data analytics [15], and new partnerships will need to be brokered when sharing data [23,24,27]. About 14% of the literature mentioned this point.

##### Lack of Appropriate Skills

It is important that health care workers are also kept up to date with the use of constantly changing technology, techniques, and a constantly moving standard of care [5,24]. Due to the constant evolution of technology, there exist populations of individuals lacking specific skills; as such this is also a significant continuing barrier to the implementation of big data [12]. About 11% of the literature expressed this challenge.

##### Inaccuracies (Veracity)

Self-reported data is extensively used in health care, and so it is crucial that the data collected in this manner be consistent [12]. Keeping information current as well as accurate is another challenge of data collection. Precision of data is also needed to provide accurate information [12]. Only 4% of the literature mentioned this challenge.

##### Regulatory Compliance Issues

Health care organizations should be aware of the various legal issues that can surface in the process of managing high volume of sensitive information. Organizations implementing big data analytics as a part of their information systems will have to comply with a significant amount of standards and regulatory compliance issues specific to health care [28]. Only 4% of the literature mentioned this challenge.

##### Real-Time Analytics (Velocity)

One of the key requirements in health care is to be able to utilize big data in real time. Real time is defined by enabling the use of applications such as cloud computing to view said data in real time. The use of these technologies leads to issues of security and privacy within patient information [12]. Only 4% of the literature mentioned this challenge. Challenges most often mentioned or discussed were data structure (17/28, 61%), security (14/28, 50%), data standardization (11/28, 39%), and data storage and transfers (8/28, 29%). The other five challenges comprised less than 15% of the observations.

#### Opportunities for Big Data in Health Care

Fourteen themes emerged under the category of opportunities: improve quality of care, managing population health, early detection of diseases, data quality, structure, and accessibility, improve decision making, cost reduction, patient-centric care, enhances personalized medicine, globalization, fraud detection, and health-threat detection. Examples of each theme are listed in Table 2. A total of 113 observations were made for opportunities.

**Table 2:** Themes that emerged from the opportunities for big data in health care.

Themes	Examples	Number of articles (n)	Articles themes appeared in	% of total articles (N=28)
Improve quality of care	Improve efficiency	18	2, 4, 5, 6, 8-13, 18-20, 22-25, 27	64%
	Improve outcomes			
	Reduce waste			
	Reduce readmissions			
	Increased productivity and performance			
	Risk reduction			
	Process optimization			
Managing population health	Managing population health	17	2, 5, 8-10, 12-14, 16, 18-20, 23, 25, 26, 28	61%
Early detection of diseases	Predicting epidemics	17	2, 4, 5, 7-13, 15, 18-20, 23, 24, 28	61%
	Disease monitoring			
	Health tracking			
	Adopt and track healthier behaviors			
	Predicting patient vulnerability			
	Improved treatments			
Data quality, structure, and accessibility	Large volumes	16	2, 4, 6, 9, 11, 12, 16, 18, 20- 23, 25-28	57%
	Wide variety			
	Creating transparency			
	High-velocity capture			
	Access to primary data			
	Reusable data			
	Weed out unwanted data			
	Open source—free access			
Improve decision making	Evidence-based medicine	11	2,-4, 7, 9, 12, 16, 20, 22, 23, 24	39%
	New treatment guidelines			
	Accuracy in information			
Cost reduction	Inexpensive	10	1, 3, 4, 7, 9, 11, 12, 14, 16, 18	36%
	Reducing health care spending			
Patient-centric health care	Empowering patients	8	2, 3, 5, 12, 14, 20, 22, 24	29%
	Patients making informed decisions			
	Increased communication			
Enhancing personalized medicine	Targeted approach	6	4-6, 24, 25, 28	24%
Globalization	Widely accessible	6	2, 6-8, 10, 20	24%
	Global sharing			
	Leveraging knowledge and practices			
	Knowledge dissemination			
Fraud detection	Fraud detection	3	8, 12, 28	11%
Health-threat detection	Health-threat detection	1	7	4%

Despite the challenges that big data needs to overcome, the advanced analytics that are promised through big data offer tremendous opportunities for most stakeholders in the health care industry (patient, provider, and payer). More than 64% of the articles analyzed focused on quality improvement and more than 60% on managing population health and early detection of diseases through big data analytics. If even some of the opportunities of big data are realized, they can radically change patient outcomes and the way decisions are made by providers, and help solve some macro-level issues related to health care within countries such as the United States (cost, quality, and access).

##### Improve Quality of Care

Big data has the potential and ability to improve the quality and efficiency of care [5,15,23,29-31]. Big data offers an ability to predict outcomes using the available primary or historical data and provide proof of benefit that could change established, industry-wide standards of care [25,28]. Leveraging technology at the patient end can also help with medication adherence [23,25]. This will most certainly play an important role in improving outcomes [2,13] and improve the health-related quality of life [20,26,32].

Quality of care will also be improved by reducing waste of information, which will reduce inefficiencies [13,26]. This will also assist in analyzing real-time resource utilization productivity [13]. Quality can also be improved by reducing the rates of readmissions, increasing operational efficiencies, and improving performance [5,12,13]. About 64% of the literature mentioned this opportunity.

##### Managing Population Health

The management of population health and the early detection of diseases were topics that the authors thought would have highly similar results after the analysis. Although there was a large overlap between the 2 themes, there was also specific variation between them. So, the researchers chose to keep them separate. The theme of managing population health focused on special populations rather than public health.

Big data analytics define populations at a finer level of granularity than has ever been previously achieved [5,14,15,33]. It can help in managing the overall health of a population as well as specific individual health [13,26,29]. Big data can enable population health management from a local or global perspective [31,34]. This capability becomes more salient from the global perspective when considering the aging of the population and age-related health issues shared by many populations and subpopulations, many of which are underserved [17,19,21,24,28,32]. About 61% of the literature mentioned this opportunity.

##### Early Detection of Diseases

Big data allows for the early detection of diseases, which aids in clinical objectives related to achieving improved treatments and higher patient outcomes [12,13,15,22,25]. It is in this area that the authors found great promise in age-related illness and disease. Along with early detection, big data analytics can also help in the prevention of a wide range of deadly illnesses and personalized disease management and monitoring [5,19,21,22,29,34]. It enables providers to track healthy behaviors and helps patients in monitoring their respective conditions [25,32,33]. This capability holds great potential when faced with either age-related diseases, or worldwide health issues such as cardiology [16,22,28,31,34]. About 61% of the literature mentioned this opportunity.

##### Data Quality, Structure, and Accessibility

Literature suggests that big data enables rapid capture of data and the conversion of primary, raw and unstructured data into meaningful information [15,17,31,34]. New knowledge can then be generated from high volumes of effective data, enabling reuse of the data [15,20,21,32,33]. Open-source technology increases accessibility to and transparency of the data [12,25,26,30,35]. Finally, data quality can be maintained using analytics to get rid of unnecessary information [27]. About 57% of the literature mentioned this opportunity.

##### Improve Decision Making

Big data enables appropriate use of evidence-based medicine and helps health care providers make more informed decisions [12,13,15,22]. This, in turn, improves the quality of care provided to the patients [16,31,36]. Remote monitoring, patient profile analytics, and genomic analytics are examples of other applications that influence the decision-making process [13,25].

Decision-making process can be highly optimized by the availability of accurate and up-to-date information, as decision making is influenced by the generation of new practices and treatment guidelines within clinical research. Allowing big data to influence decision making will allow for a faster and simpler process. This is done by either supporting or replacing human decision making. About 39% of the literature mentioned this opportunity.

##### Cost Reduction

The literature suggests that the decrease in cost of the elements of computing, such as storage and processing, leads to a decrease in the cost of data-intensive tasks [2,13]. This pass-through of savings will be seen across the spectrum of medicine [24,36] and the health care workforce [25]. Savings will be realized through more cost-effective treatments and monitoring to improve medication adherence [25,31] and through the reduction of costly transportation costs, as is experienced in cardiology [12,17,22,34]. About 36% of the literature mentioned this opportunity.

##### Patient-Centric Care

Increasing the use of technology is slowly changing the direction of the health care sector from disease-centric care toward patient-centric care [5]. Big data will play a significant role in this transformation [37]. It will allow the information to be delivered to patients directly and empower them to play an active part in their care [5,15,27]. When patients are provided with the appropriate information, it will influence their decision making and allow them to make informed decisions [13,24]. Informed decisions will also be influenced by increased communication between patients, providers, as well as their communities [5,24,32,36]. About 29% of the literature mentioned this opportunity.

##### Enhancing Personalized Medicine

With the use of big data, the objectives of personalized medicine can be translated into clinical practice [5,25,30]. Access to and processing of large volumes of data should enable a personalized patient-specific record of risks of disease [25,29,32]. Big data applications aim to make this process more efficient [12]. About 24% of the literature mentioned this opportunity.

##### Globalization

Big data will actively help in disseminating the knowledge acquired from the data collected [15,22,30]. Big data plays an active role in leveraging the practices and knowledge not only regionally but globally [12,15,29]. By globalizing data, it is made more widely accessible and providers may access new information from all regions [22,23,32]. About 24% of the literature mentioned this opportunity.

##### Fraud Detection

One of the most significant benefits offered by big data is that it is instrumental in detecting fraud in an efficient and effective manner [13,23]. For example, the unauthorized use of specific user accounts by third parties can be minimized [21]. Only about 11% of the literature mentioned this opportunity.

##### Health-Threat Detection

Big data offers opportunity for improving capabilities of threat detection quickly and more accurately. This can be especially beneficial for government use [22]. Big data augments the current acquisition of protection against the increasing threats of foreign countries, criminals, terrorists, and others. Only 3.6% of the literature mentioned this opportunity.

Opportunities most often mentioned or discussed were improve quality of care (18/28, 64%), managing population health (17/28, 61%), early detection of diseases (17/28, 60.7%), data quality structure and accessibility (16/28, 57%), improve decision making (11/28, 39.3%), cost reductions (10/28, 36%), patient-centric health care (8/28, 29%), enhancing personalized medicine (6/28, 24%), and globalization (6/28, 24%). The other two opportunities each comprised less than 15% of the observations.

## Discussion

### Summary of Evidence

Although the integration of big data is well underway in industries such as finance and advertising, it has not yet fully assimilated into health care. Challenges and opportunities were made quite clear in the articles analyzed in this review. Three of the 4 Vs (volume, velocity, and variety) were consistently adhered to. The fourth V, veracity, was found, but rarely listed by name. Tables 1 and 2 provide insightful information that is previously unpublished. These tables identify challenges and opportunities and illustrate their frequency of mention in the literature. This information is helpful to other researchers and innovators because it provides direction and proper emphasis of research effort. The listed challenges and opportunities are ordered by their frequency found in the literature.

### Limitations

A big limitation in this review is the low number of articles used in the analysis. If we were to do this over again, we would query another database to see whether additional articles were available for analysis.

Selection bias seems to exist in any study. Our control for selection bias was the initial research up front to agree on a definitive definition of the concept of big data, and our consensus meetings to discuss findings. The consensus meetings offered great value to the process because they enabled the group to hear the focus of an individual and either provide feedback to confirm the focus or agree that the unique focus was warranted for all the articles in the review.

Another bias that we discuss regularly is publication bias. Journals tend to publish results that are statistically significant, which inherently limits the publication of research that may not reach that level. Our control for publication bias was to include Google Scholar in our search. Our intent was to identify material in lesser-known journals that might not be indexed in PubMed (MEDLINE) or CINAHL.

### Conclusions

Big data and the use of advanced analytics have the potential to advance the way in which providers leverage technology to make informed clinical decisions. However, the vast amounts of information generated annually within health care must be organized and compartmentalized to enable universal accessibility and transparency between health care organizations.

Our systematic literature review revealed both challenges and opportunities that big data offers to the health care industry. The literature mentioned the challenges of data structure and security in at least 50% of the articles reviewed. The literature also mentioned the opportunities of increased quality, better management of population health, early detection of disease, and data quality structure and accessibility in at least 50% of the articles reviewed. These findings identify foci for future research.

## Appendix

Multimedia Appendix 1 Summary or relevance of cited work.

## Abbreviations

ARRA: American Recover and Reinvestment Act
EHR: electronic health record
HIE: Health Information Exchange
HIPAA: Health Insurance Portability and Accountability Act
HITECH: Health Information Technology for Economic and Clinical Health
MeSH: Medical Subject Headings
PRISMA: Preferred Reporting Items for Systematic Reviews and Meta-analysis
