# Supplementary material for: Challenges and Opportunities of Big Data in Health Care: A Systematic Review
Source: JMIR Med Inform. 2016 Nov 21;4(4):e38. doi: 10.2196/medinform.5359 (PMC5138448; doi:10.2196/medinform.5359)
Supplement: Multimedia Appendix 1 [file medinform_v4i4e38_app1.pdf]

Author	Summary or Relevance
McAfee A, Brynjolfsson E [2]	Volume: 2.5 exabytes created each day (2012), and that is doubling every 40 months.<br>Velocity: Real-time or nearly real-time information enables nearly real-time analysis of the data.<br>Variety: Disparate data sources create rich results in analysis.
Chawla NV, Davis DA [5]	Abundance of nonanalyzed data exists today. Genomic research offers more. Data mining genetic markers can provide a health-risk profile personalized to each patient.
Jee K, Kim GH [12]	Big data in health care has great potential, but current tools offer low security. The NHS (UK) free health care to permanent residents has the potential to generate vast amounts of data, and proper collection and analysis of this data can help improve the standard of care, but the public data must be merged with private data in order to capture a complete picture. South Korea owns an advanced data network, but the health disparities between urban and rural areas complicate its ability to leverage the data captured.
Raghupathi W, Raghupathi V [13]	Provides a good definition of big data, the 4Vs, and several practical examples in practice as of 2014.
Fernandes L et al [14].	Big data can be used to define patient populations at a level of granularity previously unobtainable. Big data tools can be used to find outcomes that are predicted with a high degree of sensitivity and specialty.
Beveridge R, et al [15]	Watson (of Jeopardy fame) is a very good example of “big data” in action. In health care, specifically oncology, “big data” would combine and analyze state tumor registries and claims data in order to come up with a longitudinal record—a must for proper oncology practice.
Mohr DC, et al [16]	(very little on big data) Disparities in data standards cause difficulty in analyzing multiple streams of data. More work in this area needs to be done.

Author	Summary or Relevance
Wang P, Chen Z [17]	Researchers analyzed lessons learned from studies that compare both Traditional Chinese Medicine (TCM) and Western medicine. Big data analytics revealed some insight into the field of “developing world omics.”
Hsieh JC, et al [18]	Big data in telecardiology can create a new market of e-consultations to ensure that on-site physicians are delivering appropriate treatment. Real-time consultation and tele-diagnosis of electrocardiogram and images can be practiced over an e-platform for clinical, research, and educational purposes.
Sepulveda MJ [19]	Big data causes rapid growth economies and fundamental changes in social structures and health care. Occupational and Environmental medicine is based on population health and an environmental paradigm that is changing on a rapid level. Longitudinal data collection is necessary to keep up with the changes.<br>In 2010, it was estimated that the global data generation was approximately 2.5 exabytes (2.5^{18}) per year and growing by 40% per year. One exabyte is more than 4000 times the data housed in the Library of Congress. In 2014, Twitter generated more than 7 terabytes of data per day and Facebook more than 10 terabytes per day.
Moore P, et al [20]	The aging of the population brings to the forefront the importance of the study of dementia, specifically in the areas of context and data processing to detect predictors (precursors) of dementia. The next-generation context-aware systems will most likely focus on health monitoring. Big data solutions offer noninvasive methods of caring for the aging population.

Author	Summary or Relevance
Sengupta PP [21]	Large functional datasets are created from cardiac ultrasound imaging. Cardiologists use an algorithm to help identify any of 8 echocardiographic variables in order to recognize patterns that could alter the state of care. However, these eight variables could occur, mathematically, in 40,320 different combinations (8 factorial). Cardiology is looking toward cloud-based automation and big data analysis to help the doctors improve the standard of care.
Schilsky RL, et al [23]	In 2013, the IOM suggested 6 qualities of a high-quality health care delivery system for patients with cancer. The IOM suggested the development of learning health care information technology system that enables real-time analysis of data from patients with cancer in a variety of settings. CancerLinQ was created to meet this need. It collects far more data than the other cancer registries, and its use is expected to yield advancements in care and outcomes.
Baker TB, et al [23]	It can take up to 7 years from proposal to results, which is far too long in a fast-moving technologically rich environment. Big data techniques may help health researchers manage the huge increase in technology in the last 10 years.
Kim TW, Seu JH [24]	A framework is proposed to analyze bio-signal data based on HL7 aECG standard. Hadoop experiences difficulty in analyzing unscrubbed data due to its uncommon database schema, unless using a map/reduce provided by the Hadoop platform.
Augustine DP [25]	The Indian health care system draws 1.3 million patients from abroad each year (2014), and in 2013, it generated US $3 billion. However, 99% of India's population cannot afford the services that its health care system offers to those who travel into the country for care. Big data in India can offer monitoring systems that are generating loosely structured data from different sensors that are connected to the patient over a period

Author	Summary or Relevance
	of time.
Jiang P, et al [26]	Big data has great potential to help the elderly “age in place,” which is the preference of more and more seniors in the UK. Big data installed in homes will enable the detection of falls, illnesses that cause immobility, and other unforeseen incidents.
Lamarche-Vadel A, et al [27]	Analyzed the last hospital diagnosis (International Classification of Diseases version 10) for underlying cause of death in France from 2008 to 2009 using a known algorithm comparing main diagnosis and underlying cause of death (n=421,460). The modified algorithm demonstrated mortality that was independent of diagnosis. Results could improve post-discharge care in the future.
Hrovat G, et al [28]	Researchers used an Apriori algorithm and linear-model-based recursive partitioning on over 65 million data points to identify temporal trends between otherwise unknown data points. This is a practical application of big data theory.
Howren MB, et al [29]	Age-related cognitive decline is a worldwide phenomenon—it may affect memory, orientation, attention, abstract thinning, and perception, which may lead to additional difficulties, disabilities, and limitations in everyday life. Big data analytics offers the ability to conduct widespread studies, which are necessary to determine which mental exercises will be the most effective for this population.

Author	Summary or Relevance
Issa NT, et al [30]	We are in the era of the “-omics,” wherein an individual’s genome, transcriptome, proteome, and metabolome can be scrutinized to the finest resolution to paint a personalized biochemical fingerprint that enables tailored treatments, prognoses, risk factors, and so forth. Digitization of this information parlays into “big data” informatics-driven evidence-based medical practice. While individualized patient management is a key beneficiary of next-generation medical informatics, this data also harbors a wealth of novel therapeutic discoveries waiting to be uncovered. “Big data” informatics allows for networks-driven systems pharmacodynamics whereby drug information can be coupled to cellular- and organ-level physiology for determining whole-body outcomes.
Youssef AE [31]	The data revolution is of particular interest in health care. Interoperability becomes much easier with big data techniques such as Hadoop that can analyze data whether it is structured, semi-structured, or unstructured. The analysis of disparate EMRs has the potential to find useful insights that help practitioners make critical decisions at the right time.
Wlodarczk TW, Hacker TJ [32]	Almost half of the scientific publications using predictive analytics of big data over the last 3 years have been in health care and 3 other sectors.
Kaushik K, et al [33]	Researchers proposed a framework for enhanced genetic clustering using patient history, symptoms, and existing medical conditions. Using cluster formation, cohesion and adhesion between clusters determine the present medical condition and probability for being prone to diseases in the future.
Mancini M [34]	Big data in health care is most exciting because Hadoop offers the ability to analyze both structured and unstructured data, which is a wide range in EMRs based on the vendor. Big data has the potential to discover useful information and exploit the

Author	Summary or Relevance
	potential of previously unusable data.
Song TM, et al [35]	Researchers used Google search trends as a big data analytics to identify a correlation between suicide rates and searches on suicide. The data from Google search trends contained sufficient granularity to perform a suitable analysis and data-driven conclusions.
Baro E, et al [36]	Big data requires new computational methods that optimize data management.
Naqishbandi R, et al [37]	Informational piece explaining the relationships between big data, complex event processing, and Internet of things and how the combination can be useful in health care, for example, critical care.<br>In 2010, it was estimated that the daily rate of global data generation was approximately 2.5 exabytes (2.5^{18}) of information and growing at the rate of 40% per year.
